# Suppressing Gate-Induced Drain Leakage with an Asymmetric Gate Design in HiPco CNT FETs

**DOI:** 10.3390/nano16110653

**Published:** 2026-05-22

**Authors:** Hui Ma, Senbiao Gu, Minglong Zhai, Honggang Liu

**Affiliations:** 1 School of Physics and Electronic Engineering, Shanxi University, Taiyuan 030006, China; 2 Chongqing Institute of Carbon-Based Integrated Circuits, Peking University (CICIC-PKU), Chongqing 401332, China; 3 Institute for Carbon-Based Thin Film Electronics, Peking University, Shanxi (ICTFE-PKU), Taiyuan 030012, China; 4 Center for Carbon-Based Electronics, School of Electronics, Peking University, Beijing 100871, China

**Keywords:** asymmetric gate, carbon nanotube, field-effect transistors, TCAD simulation, ultralow power

## Abstract

Carbon nanotube field-effect transistors (CNT FETs) hold great promise for extending Moore’s Law, yet their performance is critically limited by excessive off-state leakage, caused by band-to-band tunneling (BTBT) in narrow bandgap CNT channels. In this work, we overcome this long-standing bottleneck by introducing a co-design strategy that integrates a small-diameter HiPco CNT channel with a novel asymmetric gate architecture. This approach strategically reshapes the channel electrostatics to simultaneously suppress the gate-induced drain leakage (GIDL) effect and preserve excellent carrier transport. The efficacy of this strategy is rigorously validated through calibrated technology computer-aided design (TCAD) simulations for both NMOS and PMOS operation, demonstrating an ultralow off-current of 10 fA/µm, an on-current of 1.08 mA/µm, and a record on–off ratio of 1.1 × 10^11^ for back-gated CNTFETs at the 90 nm node. The design exhibits outstanding scalability: at the scaled 28 nm node with a supply voltage of 0.7 V, the PMOS device achieves 3 mA/µm on-current and 6 pA/µm off-current, maintaining an on–off ratio of 5 × 10^8^. This work establishes a scalable pathway toward femtoampere-level CNT CMOS, addressing the static power challenge in future nano-electronics.

## 1. Introduction

The relentless pursuit of Moore’s Law has driven the search for semiconductor materials that can sustain device scaling beyond silicon’s limits. Among these, semiconducting carbon nanotubes (CNTs) stand out due to their exceptional carrier mobility and near-ballistic transport, enabling field-effect transistors (FETs) with superior electrostatic control and high drive currents at aggressively scaled nodes [1,2,3,4,5,6,7,8,9,10,11]. This has led to the experimental demonstration of high-performance CNT FETs with gate lengths as short as 5 nm, validating their potential for ultimately scaled electronics [11].

Current state-of-the-art CNTFETs mainly employ single-walled carbon nanotubes (SWCNTs) with a bandgap *E_g_* ≤ 0.6 eV. While these devices often report excellent on-state currents exceeding 1 mA/µm at a drain-source bias of −0.5 V, the corresponding off-state leakage is rarely mentioned. Narrow-bandgap CNT FETs typically present off-state currents above 100 nA/µm, significantly exceeding the sub-10 nA/µm benchmark of modern silicon technology [12,13,14,15,16,17]. Importantly, when the operating voltage rises above 1.0 V, this leakage grows sharply. The behavior is dominated by the combined effects of band-to-band tunneling (BTBT) and Schottky-barrier tunneling at the metal-CNT contacts [18,19]. These two tunneling mechanisms create a double-barrier effect, which makes off-state leakage difficult to control. This poses a significant challenge for practical implementation and energy-efficient digital integration.

Researchers face several technical challenges in tackling the issue of leakage current. Firstly, doping-based electrostatic control is unviable, as the LDD approach—essential in silicon—is precluded by the doping-resistant sp^2^ lattice of CNTs. Secondly, L-shaped contact or multi-gate designs can modulate the leakage, their structural complexity introduces severe integration challenges, parasitic penalties, and ultimately a hard scalability barrier below 50 nm [20,21]. Thirdly, the tunneling suppression achieved by using small-diameter CNTs comes at the cost of a widened bandgap, which reduces the density of states and carrier injection efficiency, potentially limiting the maximum on-current [22]. Consequently, achieving ultralow off-state currents while maintaining a steep subthreshold swing (SS < 60 mV/dec) remains a central challenge.

To break this fundamental limitation, we introduce a co-design strategy that integrates a high-purity, small-diameter HiPco CNT channel with a novel asymmetric gate architecture. In contrast to conventional approaches that rely on drain engineering or feedback-gate structures to suppress short-channel effects via drain-side electrostatic modulation, this proposed strategy is dominated by source-side barrier control. By enhancing the electrostatic coupling between the gate and the source, the source-side gate control capability is strengthened. As a result, the barrier height in the off state becomes more stable. Meanwhile, the influence of the drain electric field on the barrier is significantly weakened. This attenuation suppresses thermionic emission and diminishes drain-induced barrier lowering (DIBL). The efficacy of this strategy is rigorously validated through calibrated technology computer-aided design (TCAD) simulations for both NMOS and PMOS operation, demonstrating breakthrough performance. At the 90 nm node, the proposed device achieves an ultralow off-state current of 10 fA/µm, a high on-state current of 1.08 mA/µm, and a record on–off ratio of 1.1 × 10^11^. Furthermore, the design exhibits outstanding scalability. At the scaled 28 nm node with a supply voltage of 0.7 V, the PMOS device delivers an on-current of 3 mA/µm and an off-current of 6 pA/µm, maintaining an on–off ratio of 5 × 10^8^. The NMOS exhibits the same performance, demonstrating excellent symmetry with the PMOS. This work provides a critical solution to the static power challenge.

## 2. Experiment

**Figure 1:**
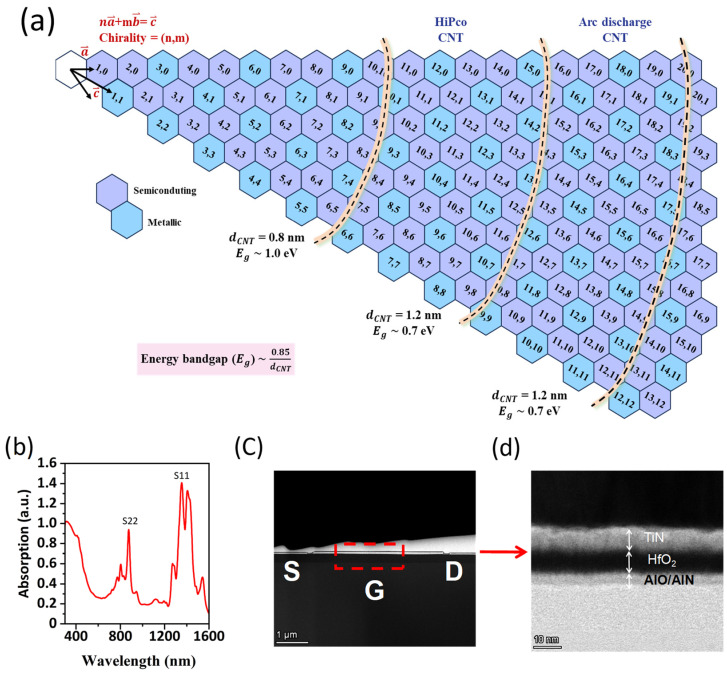
(**a**) Chirality table for single-walled carbon nanotubes. The primary diameter ranges for HiPco CNTs and Arc discharge CNTs are indicated in the plot [23]. (**b**) Absorption spectrum of HiPco CNTs. (**c**) Cross-sectional HAADF-STEM image of the fabricated top-gate field-effect transistor. The red dashed box marks the gate (G) region, with source (S) and drain (D) labeled on both sides. Scale bar: 1 μm. (**d**) Cross-sectional BF-TEM image of the gate stack, showing the 4 nm HfO_2_ and 2 nm AlON layered structure.

To validate our simulation framework, we fabricate top-gated p-type CNT FETs with a 90 nm gate length. Figure 1c shows a cross-sectional high-angle annular dark-field (HAADF) scanning transmission electron microscopy (STEM) image of the device structure. We employ high-purity semiconducting single-walled carbon nanotubes (s-SWCNTs) with a relatively large bandgap of 0.71~0.85 eV, derived from HiPco CNTs (small-diameter: 1.0–1.2 nm), as shown in Figure 1a [23]. The CNT spectral plot is shown in Figure 1b. The channel is formed by a network of HiPco CNTs with a density of approximately 30 CNTs/µm. Palladium (Pd) source/drain contacts are placed 50 nm from the gate edge. The gate stack consisted of a TiN with a 4 nm HfO_2_/2 nm AlON dielectric bilayer, where AlON served as a passivation layer. Figure 1d displays the cross-sectional bright-field (BF) TEM microstructure of the gate stack. The detailed preparation process is provided in the Appendix A.

**Figure 2:**
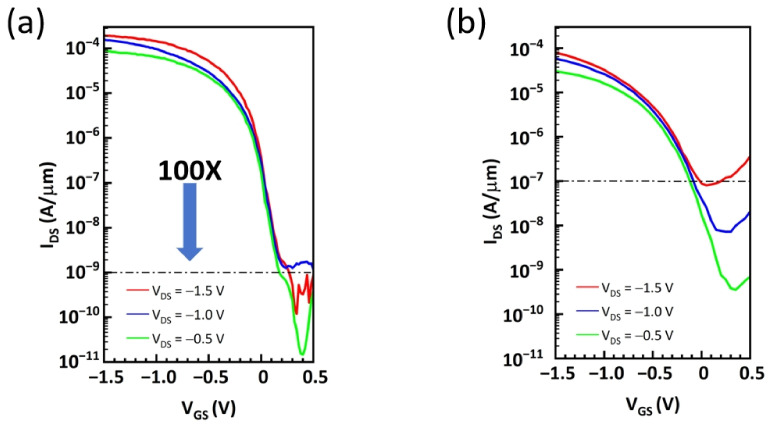
Measured transfer characteristics under different V_DS_ for symmetric 90 nm top-gate PMOS devices with (**a**) a small-diameter HiPco CNT channel and (**b**) a large-diameter arc-discharge CNT channel [24].

The measured transfer characteristics of the fabricated devices are shown in Figure 2a. Compared with a narrow-bandgap (~0.56 eV) CNT device shown in Figure 2b [24], the HiPCo CNT PMOS exhibits nearly two orders of magnitude lower off-state while delivering a high on-state current of ~200 µA/µm at V_DS_ = −1.5 V. The off-state leakage remains below 1 nA/µm with week dependence on V_DS_, indicating effective suppression of the gate-induced drain leakage (GIDL). These experimental results confirm the advantage of using small-diameter semiconducting CNTs for low-leakage transistor applications.

## 3. Results and Discussion

### 3.1. Simulation Methodology

Our physical models and parameters were calibrated against the experimental data of the fabricated device (Figure 1c). All simulations were performed using the two-dimensional (2D) Sentaurus TCAD platform. Carrier transport in the CNT channel was modeled using the classical drift–diffusion (DD) framework. To account for off-state leakage at the drain side, a nonlocal band-to-band tunneling (BTBT) model was employed along the transport direction. Carrier mobility in the CNT channel was modeled using the Sentaurus Mobility module, which includes field-dependent mobility (Enormal), high-field velocity saturation, the Caughey–Thomas low-to-high-field transition, and quasi-Fermi gradient effects [25]:

(1) μn,p=μlow11+μlowvsat n,pEβ1β

where $\mu_{low}$ denotes the low-field electron (hole) mobility. E is the parallel electric field. *v*_sat n,p_ represents the electron (hole) saturation velocity in high fields, and *β* is a fitting parameter. This formulation accounts for both low-field and high-field transport while incorporating the influence of carrier density variations. Carrier injection at the source and drain contacts was treated using a semiclassical tunneling model based on the WKB approximation, which captures both thermionic emission and tunneling transport across the metal–CNT interfaces. The effect of interface trap states at the gate dielectric interface was included using an interface trap model. The carrier concentration in semiconducting HiPico CNTs was calculated under nondegenerate conditions using a one-dimensional density-of-states model that accounts for the Van Hove singularities at the band edges. The effective density of states is expressed as [26,27]:

(2) $$N_{C}\approx \frac{g_{0}}{4}\sqrt{\pi kTE_{g}}$$

where $g_{0}={2\;nm}^{-1}{eV}^{-1}$ is the CNT material constant, k is the Boltzmann constant, T is the temperature, and *E_g_* is the CNT bandgap. The carrier concentration is then given by Equation (3). The effective mass m* of the CNT is taken as Equation (4) [26,27].

(3) $$n=2N_{c}exp\left(\frac{E_{F}-E_{C}}{kT}\right)$$

(4) $$m^{*}=\frac{{4\hbar }^{2}E_{g}}{3\gamma a^{2}\left(2\gamma +E_{g}\right)}$$

where γ = 3.1 eV is the nearest-neighbor overlap energy. ℏ is the reduced Planck’s constant and a = 2.46 Å is the lattice constant.

**Figure 3:**
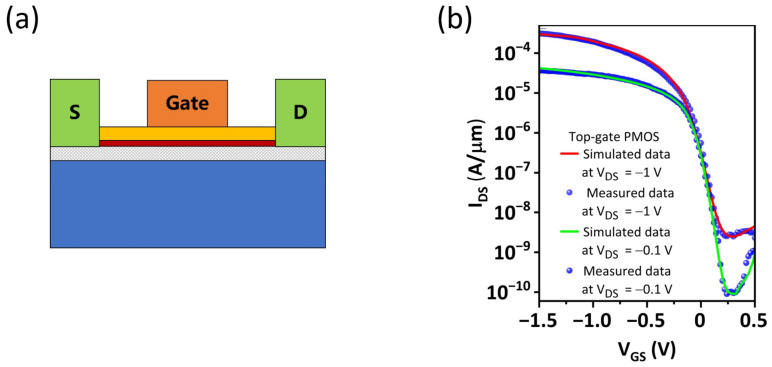
(**a**) Schematic of the self-aligned top-gate HiPco CNT PMOS structure, featuring a 90 nm gate length, 6 nm AlON/HfO2 gate oxide (red: AlON, yellow: HfO_2_). S and D denote the Pd source and drain electrodes, and the bottom blue region is the silicon substrate. (**b**) Measured (blue dots) and simulated (red/green lines) transfer characteristics for the 90 nm top-gate HiPco CNT PMOS devices at V_DS_ = −1.0 V and −0.1 V.

Using the above physical models and parameters (Table 1), we constructed a TCAD device model. The model is calibrated using the 90 nm top-gate CNT-FET measurements (Figure 2a), the simulated results show excellent agreement with experiments, as shown in Figure 3a, validating the model’s accuracy. The calibration primarily targets intrinsic CNT transport and metal–CNT contact physics, which are material-dependent and therefore transferable across different device geometries and stack configurations. Leveraging this model, we explored the design space of 130 nm back-gated CNT PMOS devices shown in Figure 3b. The source/drain contacts are defined with Pd, while the gate control was achieved with an HfO_2_ dielectric, and the device stability was ensured by an AlON passivation layer.

**Figure 4:**
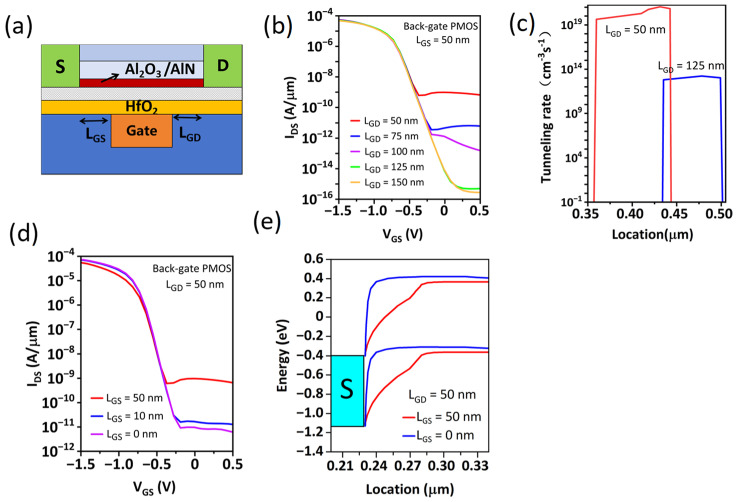
(**a**) Schematic of a back-gate HiPco CNT NMOS with a 130 nm gate length, 50 nm spacer, and HfO_2_ gate oxide. The double-headed arrows mark the key length parameters of the device: L_GS_ represents the Gate-to-Source distance, and L_GD_ represents the Gate-to-Drain distance. (**b**) Simulated transfer characteristics for back-gate CNT PMOS with varying L_GD_ biased at V_DS_ = −1.0 V. (**c**) Tunneling rate near the drain for different L_GD_ values at V_DS_ = 0 V. (**d**) Simulated transfer characteristics for back-gate CNT PMOS with varying L_GS_ biased at V_DS_ = −1.0 V. (**e**) Energy band diagram near the source region of devices biased at V_DS_ = 0 V.

We conducted a systematic investigation into the geometric determinants of off-state leakage, aiming to establish clear guidelines for optimizing CNT FET performance. Adjusting the Gate-to-Drain (L_GD_) and Gate-to-Source (L_GS_) spacing provides an effective means to mitigate off-state leakage by modulating electric field distributions. Increasing L_GD_ under a fixed L_GS_ of 50 nm introduces an extended electric field buffer region. This buffer attenuates the drain-field penetration into the gated channel, thereby significantly weakening the drain-induced barrier lowering (DIBL) effect and suppressing the associated tunneling current. Quantitatively, extending L_GD_ to 125 nm reduces the off-state leakage by up to five orders of magnitude shown in Figure 4a. Figure 4b shows the tunneling probability near the drain for different L_GD_ values while the device is in the off-state.

Conversely, in a back-gated architecture with fixed L_GD_ of 50 nm, reducing L_GS_ strongly enhances the gate’s electrostatic control over the source-side channel potential. This short-range coupling suppresses the lateral spread of the DIBL effect near the source. As shown in Figure 4c, by minimizing L_GS_ from 50 nm to 0 nm, achieves a current leakage reduction of three orders of magnitude. The corresponding band diagram is shown in Figure 4d. When the LGS is shortened, the potential barrier at the source end is raised and becomes more stable. This higher barrier raises the energy threshold for thermionic emission. As a result, carrier injection in the off-state is drastically reduced. At the nanoscale, the relative placement of the gate with respect to the source and drain becomes a critical factor.

### 3.2. Geometric Effects on Off-State Leakage

**Figure 5:**
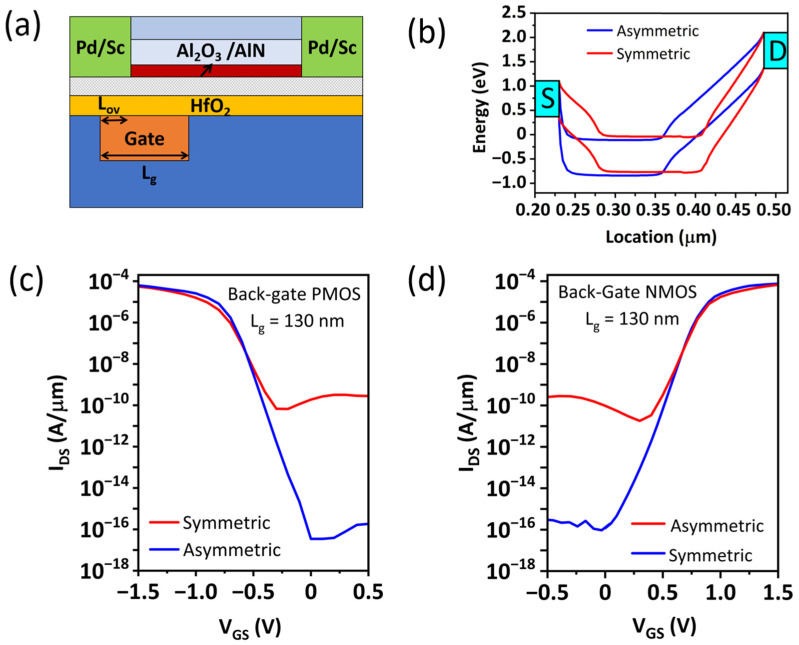
(**a**) Schematic illustrating the proposed asymmetric back-gated HiPco CNT FETs structure. The double-headed arrows mark the key length parameters of the device: L_g_ represents the physical gate length, and L_ov_ represents the gate-to-source overlap length. (**b**) Energy band diagram of the asymmetric and symmetric PMOS device. (**c**) Comparison of transfer characteristics for the asymmetric and symmetric HiPco CNT PMOS devices under V_DS_ = −1.0 V. (**d**) Comparison of transfer characteristics for the asymmetric and symmetric HiPco CNT NMOS devices under V_DS_ = 1.0 V.

We propose an asymmetric back-gated CNT PMOS(NMOS) architecture integrating high-purity small diameter HiPco CNT channels. The channel in both types of devices is formed by a network of HiPco CNTs with a density of approximately 30 CNTs/µm. The Source and Drain metals differ between the two devices, with Scandium (Sc) in NMOS and Palladium (Pd) in PMOS. The gate metal stack consisted of a TiN with a 5 nm HfO_2_ dielectric bilayer, where AlON served as a passivation layer. As illustrated in Figure 5a, the gate is positioned adjacent to the source. Based on the above device structure, we perform a symmetry analysis on devices with a gate length (L_g_) of 130 nm and a total channel length (L_ch_) of 230 nm, in order to evaluate the impact of different gate placements on device performance. This design suppresses the off-state current by around five orders of magnitude in both NMOS and PMOS devices as shown in Figure 5c,d. The underlying physical mechanism is elucidated in Figure 5b. Firstly, it raises and stabilizes the carrier injection barrier at the source, thereby limiting the initial carrier influx into the channel. Secondly, it extends and broadens the potential barrier near the drain. Notably, the asymmetric devices exhibit a reduced subthreshold swing, from approximately 85 mV/dec for the symmetric devices to ~64 mV/dec, indicating enhanced gate control and a corresponding improvement in switching speed.

**Figure 6:**
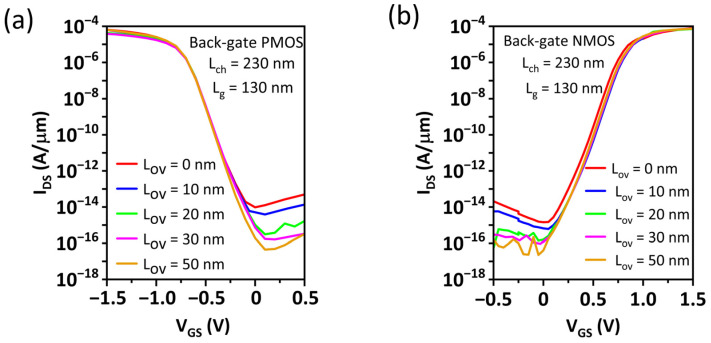
(**a**) Simulated transfer curves for PMOS with the gate positioned at different locations beneath the source, under V_DS_ = −1.0 V. (**b**) Simulated transfer curves for NMOS with the gate positioned at different locations beneath the source, under V_DS_ = 1.0 V.

To evaluate the sensitivity of placing the gate near the source, we define the gate-source overlap length (L_OV_) as the physical distance between the gate edge and the source side of the channel. Subsequent simulations focused on its effect on device performance. Figure 6a,b show the transfer curves. When L_OV_ exceeds 20 nm, the reduction in off-state leakage becomes marginal. The results indicate that the device enters a field-controlled saturation regime. This regime is characterized by the fact that further increasing the L_OV_ neither significantly enhances electrostatic control near the source. In practical device fabrication, small deviations in gate placement within this range of L_OV_ have a limited impact on performance. This indicates that the design is tolerant to placement variations and provides a key reference for future device iterations.

To clearly illustrate the combined effect of CNT diameter and gate architecture on the off-state current (Ioff), We design three sets of comparative simulations. In these simulations, all key parameters, including L_g_, L_ch_, V_DS_, and dielectric stack, were kept constant. Only the CNT diameter and gate symmetry were varied to isolate their respective effects. The simulation results are summarized in Table 2. Reducing the CNT diameter alone lowers the off-state current by roughly two orders of magnitude. This reduction is mainly caused by the larger bandgap that small-diameter CNTs possess. The widened bandgap effectively suppresses both thermionic and tunneling leakage. On this basis, introducing an asymmetric gate further reduces the leakage current by more than five orders of magnitude, indicating that the asymmetric gate can modulate the electrostatic field distribution to further suppress carrier leakage and significantly enhance off-state performance.

**Figure 7:**
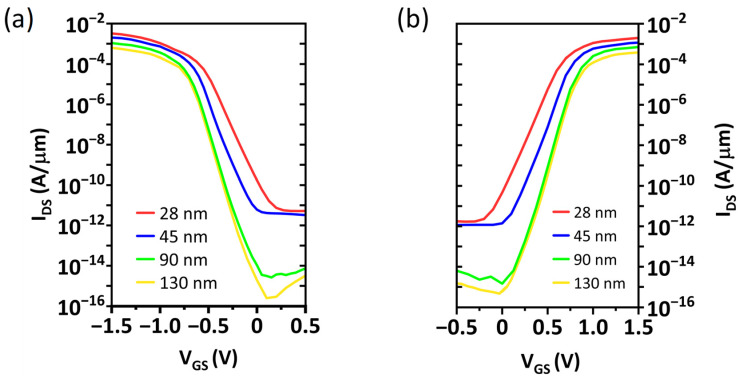
Simulated transfer characteristics comparison for proposed (**a**) PMOS and (**b**) NMOS devices with varying gate lengths (28 nm and 45 nm at V_DS_ = 0.7 V; 90 nm and 130 nm at V_DS_ = 1.0 V).

To quantitatively assess the scalability of proposed strategy, we further develop a model of carbon-nanotube (CNT) array transistors. Device currents per unit width are normalized to a CNT line density of ~125 CNTs µm^−1^ [21], and we simulate the electrical characteristics of asymmetric back-gated CNT field-effect transistors (FETs) of both p-type and n-type, for gate lengths ranging from 28 nm to 130 nm. The device geometries at different technology nodes were obtained by geometric scaling from a 130 nm reference structure, where all characteristic lengths (L_ch_, L_OV_, and L_GD_) were scaled proportionally by the same factor, while the intrinsic CNT transport and contact parameters were kept unchanged. The key scaled parameters and corresponding simulation results for each node are summarized in Table 3. Figure 7a,b plot the transfer characteristics. At the aggressively scaled 28 nm node (with corresponding L_OV_ = 6 nm and L_GD_ = 30 nm), the device maintains an on/off ratio greater than 10^8^ and an SS of ~85 mV/dec.

## 4. Conclusions

An empirically calibrated TCAD framework is developed for accurate simulation of back-gated small-diameter CNTFETs. Using this framework, the geometric origins of off-state leakage, particularly gate-induced drain leakage are analyzed. A source-aligned asymmetric gate design effectively suppresses short-channel effects by reshaping the channel electrostatics, leading to more than five orders of magnitude reduction in off-state current compared with conventional symmetric gates at a gate length of 130 nm. The proposed structure shows excellent scalability, maintaining an on/off ratio above and a near-ideal subthreshold swing (~85 mV/dec) at a gate length of 28 nm, indicating a viable path toward femtoampere-level CNT CMOS technology.

## Figures and Tables

**Table 1:** Parameters used for model calibration.

Parameter	HiPco CNT PFET
Gate length (L_g_)	90 nm
Gate oxide thickness (T_ox_)	6 nm
Diameter (d_CNT_)	1.15 nm
Bandgap (E_g_)	0.74 eV
Affinity (eV)	4.1 eV
Effective mass (m*)	0.06 m_0_
Mobility of channel (μ_0_)	$1\times 10^{4}$ cm^2^/(V⋅s)
Effective density of states (Nc)	$0.24\times 10^{5}$ cm^−1^
Saturation velocity (v_sat_)	$1.1\times 10^{7}$ cm/s
Contact work function ($\phi_{m}$)	5.1 eV
Gate work function ($\phi_{G}$)	5.1 eV

**Table 2:** Benchmarks of 130 nm L_g_ CNT PMOS with different device structures under V_DS_ = −1 V.

Structure	d_CNT_ (nm)	E_g_ (eV)	I_off_ (A/μm)	I_on_/I_off_
Symmetric gate	1.5	~0.56	5.2 × 10^−9^	2.4 × 10^4^
Symmetric gate	1.1	~0.74	6.8 × 10^−11^	8 × 10^5^
Asymmetric gate	1.1	~0.74	3.4 × 10^−17^	1.7 × 10^12^

**Table 3:** Scaled parameters of each node with corresponding simulation results.

L_g_ (nm)	L_ov_ (nm)	L_GD_ (nm)	L_ch_ (nm)	T_ox_ (nm)	I_on_/I_off_	SS (mV/dec)
28	6	30	52	3.5	10^8^	85
45	10	60	95	5	10^8^	78
90	20	120	190	5	10^11^	65

## Data Availability

The original contributions presented in this study are included in the article. Further inquiries can be directed to the corresponding author.

## Appendix A

### Appendix A.1. Fabrication of Gate Stack and PMOS Device

[I] Construction of Gate Stack on 8-inch N-type Silicon Wafer

1. Deposition of High-purity Semiconductor Carbon Nanotube Network

A network of small-diameter carbon nanotubes (CNTs) is first deposited on an 8-inch N-type silicon (Si) wafer substrate. To achieve this, polymer-based dispersion and purification techniques are employed: through multiple cycles of polymer dispersion and purification, a super-pure semiconductor CNT solution is obtained. This process elevates the purity of semiconductor CNTs to 99.99995%, thereby minimizing the adverse effects of metallic CNTs on subsequent device performance, such as leakage and inhomogeneity. Subsequently, the solution is deposited onto the silicon wafer surface using the static sedimentation method, forming a uniform and dense network-like CNT film through solvent evaporation.

2. High-k Gate Stack Fabrication by Atomic Layer Deposition

A complete gate stack structure is formed on the carbon nanotube film by sequentially growing aluminum oxynitride (AlON), hafnium oxide (HfO_2_) and titanium nitride (TiN) via atomic layer deposition (ALD) technology.

2 nm AlON buffer layer: Initially, aluminum oxide (Al_2_O_3_) is deposited, followed by in situ nitridation to form AlON. This thin layer serves as a buffer, optimizing the interface properties between carbon nanotubes and high-k dielectrics while reducing the density of interfacial states.

The 4 nm HfO_2_ gate dielectric layer: As the core gate dielectric layer, HfO_2_ delivers enhanced gate control capability while maintaining physical thickness due to its high dielectric constant (high k value), effectively suppressing leakage current caused by quantum tunneling effects.

10 nm TiN metal gate layer: The ALD-deposited TiN serves as the metal gate electrode, featuring an optimal work function and excellent chemical stability, enabling effective contact with HfO_2_.

The ALD technique ensures precise thickness control and excellent step coverage at the atomic scale, which is essential for forming uniform, pinhole-free laminations on complex carbon nanotube network surfaces.

3. PDA (Post-Diffusion Annealing) treatment

The prepared gate stack wafer was annealed in a nitrogen (N_2_) atmosphere at 400 °C. This annealing process effectively eliminates volume defects and interface trap charges introduced during ALD, repairs dangling bonds, and significantly improves the insulation quality and reliability of the HfO_2_ gate dielectric, thereby reducing gate leakage current.

[II] Fabrication Process of PMOS Devices

1. Sample Cleaning and Pre-treatment

After cutting the wafer prepared by the gate stack process into small pieces, one sample is subjected to rigorous wet cleaning. The cleaning sequence proceeds as follows: rinsing with deionized water (DIW) to dissolve water-soluble ionic impurities; acetone immersion to remove organic residues such as photoresist and oils; isopropanol (IPA) rinsing to eliminate acetone residues and particulate contaminants; and ethanol final rinsing that utilizes its rapid evaporation for water-free drying. Post-cleaning, the sample is baked on a 120 °C hot plate for 5 min to completely remove surface adsorbed moisture and solvents. This multi-step cleaning protocol ensures atomic-level surface cleanliness of the wafer, which serves as the foundation for all subsequent micro-nano processing steps and effectively prevents pattern defects and electrical performance degradation caused by contaminants.

### Appendix A.2. Mark and Gate Pattern Definition

A poly(methyl methacrylate) (PMMA) electron beam resist is spin-coated onto the clean sample, and alignment marks and gate patterns are precisely defined using electron beam lithography (EBL). After development and fixation, a 5/70 nm titanium/gold (Ti/Au) metal layer is deposited via thermal evaporation. Finally, the sample is immersed in acetone for lift-off to remove PMMA and excess metal, leaving the precise metal pattern.

2. The Complete Corrosion of TiN Metal Gate

The sample was subjected to wet etching using SC1 standard cleaning solution (ammonia: hydrogen peroxide: water = 1:2:10) to completely remove the topmost TiN layer. This solution exhibits exceptional etching selectivity for HfO_2_, effectively removing TiN while perfectly preserving the underlying HfO_2_ gate dielectric layer. The etching process was maintained for 4 min, after which a probe station (MPI Corporation, Hsinchu, Taiwan, China) and semiconductor parameter analyzer (Keithley Instruments, Cleveland, OH, USA) were employed to verify the complete removal of TiN.

By leveraging the high selectivity of SC1 solution for TiN and HfO_2_, precise and non-destructive removal of TiN was achieved. This step is critical as it aims to eliminate unintentional bridging or leakage paths between the gate and source/drain regions that may result from residual TiN.

3. Definition of Active Region and Graphicalization of Carbon Nanotube Channel

After patterning the active region via EBL, reactive ion etching (RIE) is employed to remove excess carbon nanotube films and the overlying gate dielectric outside the active region (i.e., the isolation region). The etching process is conducted in three steps: First, dry etching of exposed HfO_2_ is performed using SF_6_/Ar/O_2_ mixed gas; then, wet etching of exposed AlON layers is carried out with MF319 developer (a diluted alkaline solution based on tetramethylammonium hydroxide (TMAH)); finally, the bottommost carbon nanotubes are removed by plasma ashing using O_2_. This combined dry RIE and wet etching strategy achieves efficient and selective removal of different material layers, effectively isolating individual devices and eliminating leakage and short-circuit risks caused by carbon nanotube networks or dielectric layers.

4. The activation of the source-drain contact region (S/D region)

Using EBL to define the source/drain regions, the HfO_2_ layer in these areas is etched away using RIE (same SF_6_/Ar/O_2_ atmosphere). Subsequently, wet etching with MF319 solution selectively removes the AlON layer above the carbon nanotubes, exposing the carbon nanotube channel material in the source/drain regions. Precisely opening the source/drain contact window is critical for forming high-quality source/drain electrodes. The high selectivity of MF319 for AlON ensures the carbon nanotubes remain undamaged, creating an ideal surface for subsequent formation of high-quality metal-semiconductor contacts.

5. Ultraviolet Ozone (UVO) Surface Treatment

After opening the contact window, the sample is placed into the ultraviolet ozone (UVO) cleaning device for processing. UVO treatment effectively removes residual EBL adhesive while slightly functionalizing the surface of carbon nanotubes, improving their surface hydrophilicity. This enhances the film quality during subsequent metal deposition and reduces contact resistance.

6. Preparation of Source-Leakage Electrode

Palladium (Pd) metal is deposited on the source/drain regions of carbon nanotubes (CNTs) using electron beam evaporation (EBE) equipment (Vikaitech, Suzhou, China). With a high work function (approximately 5.1 eV), Pd forms excellent ohmic contact with the valence band of CNTs, making it an ideal electrode material for P-type MOSFETs. After coating, the excess EBE adhesive and surface Pd are removed through lift-off process.

The MOS structure device based on carbon nanotubes is prepared.

## References

[1] Antoniadis D.A., Aberg I., Chleirigh C.N., Nayfeh O.M., Khakifirooz A., Hoyt J.L. (2006). Continuous MOSFET performance increase with device scaling: The role of strain and channel material innovations. IBM J. Res. Dev..

[2] Thompson S.E., Parthasarathy S. (2006). Moore’s law: The future of Si microelectronics. Mater. Today.

[3] Yeric G. Moore’s law at 50: Are we planning for retirement?. Proceedings of the 2015 IEEE International Electron Devices Meeting (IEDM).

[4] Chau R., Doyle B., Datta S., Kavalieros J., Zhang K. (2007). Integrated nanoelectronics for the future. Nat. Mater..

[5] Peng L.M., Zhang Z.Y., Wang S. (2014). Carbon nanotube electronics: Recent advances. Mater. Today.

[6] Zhang Z.Y., Wang S., Wang Z.X., Ding L., Pei T., Hu Z.D., Liang X.L., Chen Q., Li Y., Peng L.M. (2009). Almost perfectly symmetric SWCNT-based CMOS devices and scaling. ACS Nano.

[7] Peng L.M., Zhang Z.Y., Qiu C.G. (2019). Carbon nanotube digital electronics. Nat. Electron..

[8] Lee C.S., Pop E., Franklin A.D., Haensch W., Wong H.S.P. (2015). A compact virtual-source model for carbon nanotube FETs in the sub-10-nm regime—Part I: Intrinsic elements. IEEE Trans. Electron Devices.

[9] Xu L., Qiu C.G., Zhao C.Y., Zhang Z.Y., Peng L.M. (2019). Insight into ballisticity of room-temperature carrier transport in carbon nanotube field-effect transistors. IEEE Trans. Electron Devices.

[10] Qiu C.G., Zhang Z.Y., Xiao M.M., Yang Y.J., Zhong D.L., Peng L.M. (2017). Scaling carbon nanotube complementary transistors to 5-nm gate lengths. Science.

[11] Franklin A.D. (2015). Nanomaterials in transistors: From high-performance to thin-film applications. Science.

[12] Lin Y., Cao Y., Lu H., Liu C., Zhang Z., Jin C., Peng L.-M., Zhang Z. (2023). Improving the performance of aligned carbon nanotube based transistors by refreshing the substrate surface. ACS Appl. Mater. Interfaces.

[13] Lin Y., Cao Y., Ding S., Zhang P., Xu L., Liu C., Hu Q., Jin C., Peng L.-M., Zhang Z. (2023). Scaling aligned carbon nanotube transistors to a sub-10 nm node. Nat. Electron..

[14] Lin Y., Liang S., Xu L., Liu L., Hu Q., Fan C., Liu Y., Han J., Zhang Z., Peng L.-M. (2022). Enhancement-mode field-effect transistors and high-speed integrated circuits based on aligned carbon nanotube films. Adv. Funct. Mater..

[15] Liu L., Han J., Xu L., Zhou J., Zhao C., Ding S., Shi H., Xiao M., Ding L., Ma Z. (2020). Aligned, high-density semiconducting carbon nanotube arrays for high-performance electronics. Science.

[16] Li S., Chao T.-A., Gilardi C., Safron N., Su S.-K., Zeevi G., Bechdolt A., Passlack M., Oberoi A., Lin Q. High-performance and low parasitic capacitance CNT MOSFET: 1.2 mA/μm at V DS of 0.75 V by self-aligned doping in sub-20 nm spacer. Proceedings of the 2023 International Electron Devices Meeting (IEDM).

[17] Yeap G., Lin S.S., Chen Y.M., Shang H.L., Wang P.W., Lin H.C., Peng Y.C., Sheu J.Y., Wang M., Chen X. 5 nm CMOS production technology platform featuring full-fledged EUV, and high mobility channel FinFETs with densest 0.021 μm^2^ SRAM cells for mobile SoC and high performance computing applications. Proceedings of the 2019 IEEE International Electron Devices Meeting (IEDM).

[18] Lin Q., Pitner G., Gilardi C., Su S.-K., Zhang Z., Chen E., Bandaru P., Kummel A., Wang H., Passlack M. (2022). Bandgap extraction at 10 K to enable leakage control in carbon nanotube MOSFETs. IEEE Electron Device Lett..

[19] Lin Q., Gilardi C., Su S.-K., Zhang Z., Chen E., Bandaru P., Kummel A., Radu I., Mitra S., Pitner G. (2023). Band-to-band tunneling leakage current characterization and projection in carbon nanotube transistors. ACS Nano.

[20] Qiu C.G., Zhang Z.Y., Zhong D.L., Si J., Yang Y.J., Peng L.M. (2015). Carbon nanotube feedback-gate field-effect transistor: Suppressing current leakage and increasing on/off ratio. ACS Nano.

[21] Xu L., Qiu C.G., Peng L.M., Zhang Z.Y. (2021). Suppression of leakage current in carbon nanotube field-effect transistors. Nano Res..

[22] Su S.K., Chen E., Hung T.Y.T., Li M.Z., Pitner G., Cheng C.C., Wang H., Cai J., Wong H.S.P., Radu I.P. Perspective on low-dimensional channel materials for extremely scaled CMOS. Proceedings of the 2022 IEEE Symposium on VLSI Technology and Circuits (VLSI).

[23] Chiu H.-Y., Safron N., Passlack M., Chao T.-A., Su S.-K., Mao P.-S., Chou C.-H., Huang H.-Y., Wu G.-Z., Chen C.-W. (2025). Overcoming the leakage and contact resistance challenges in highly scaled PMOS and NMOS carbon nanotube transistors. Nano Lett..

[24] Liu L.J., Zhao C.Y., Ding L., Peng L.M., Zhang Z.Y. (2020). Drain-engineered carbon-nanotube-film field-effect transistors with high performance and ultra-low current leakage. Nano Res..

[25] Caughey D.M., Thomas R.E. (1967). Carrier mobilities in silicon empirically related to doping and field. Proc. IEEE.

[26] Akinwande D., Nishi Y., Wong H.S.P. (2008). An analytical derivation of the density of states, effective mass, and carrier density for achiral carbon nanotubes. IEEE Trans. Electron Devices.

[27] Liang J.L., Akinwande D., Wong H.S.P. (2008). Carrier density and quantum capacitance for semiconducting carbon nanotubes. J. Appl. Phys..

